# School non-attendance and learned helplessness: latent profiles and ROC curves

**DOI:** 10.3389/fpsyg.2025.1557915

**Published:** 2025-06-18

**Authors:** María Pérez-Marco, Andrea Fuster, Carolina Gonzálvez

**Affiliations:** Department of Developmental Psychology and Didactics, Faculty of Education, University of Alicante, San Vicente del Raspeig, Spain

**Keywords:** learned helplessness, school non-attendance, adolescents, latent profile analysis, ROC curves

## Abstract

Due to the complex school reality, Learned Helplessness (LH) is a student’s response characterized by lack of confident, interpretative bias and negative outlook of success in face of school challenges. These helpless students develop a negative attitude toward school, leading to a withdrawal of school engagement and anxious disorders, reporting links with emotionally based school non-attendance. Taking into account the heterogeneous causes of these problems, in recent years new instruments have been emerged, like *Assessing Reasons for School Non-Attendance* (ARSNA; Havik et al., 2015), of which there is a clear lack of research. The study aims to: (1) identify latent profiles of school absenteeism based on *Assessing Students’ Reported Reasons for School Non-attendance* (ARSNA; Havik et al., 2015); (2) analyze differences between school non-attendance profiles and Learned Helplessness (LH); and (3) establish the predictive and discriminative capacity of LH to identify students of the high school non-attendance profile. Consequently, 759 adolescents (*M* = 14.95, *SD* = 1.82) fulfilled ARSNA (Havik et al., 2015) and *Learned Helplessness Questionnaire* (LHQ; Sorrenti et al., 2015). Pearson’s correlation coefficients reported positive and statistically significant correlations between ARSNA dimensions and LH. Latent Profile Analyses revealed 3 school absenteeism profiles. ANOVA indicated statistically significant differences between these profiles and LH. Finally, Logistic Regression and ROC Curves found the predictive and discriminative ability of LH to identify individuals of the high school non-attendance profile. Results contribute to the literature on ARSNA dimensions and LH, highlighting the potential implications for schools and for the intervention against emotionally based school non-attendance.

## 1 Introduction

Due to the massive competitiveness of education systems and the difficulty of today’s societies, many students evidence emotional problems and different ways of coping with school complexities (Raufelder et al., 2017). Among these, Diener and Dweck (1980) classified the students’ responses into Mastery-Orientation (MO), related to motivation and persistence to work harder against failure, and Learned Helplessness (LH), characterized by lack of confident, interpretative bias and negative outlook of success (Filippello et al., 2020).

Specifically, LH emerges as a psychological pattern of attributing failures to internal, stable and broad personal factor, while successes are attributed to external, changeable and specific factors (Abramson et al., 1989; Diener and Dweck, 1980; Filippello et al., 2020; Seligman and Maier, 1967). These helpless students tend to avoid challenging tasks or select easier ones to minimize the risk of failure, because they see it as confirmation of their perceived lack of ability (Raufelder et al., 2017). Additionally, they do not attach any value to engagement, believing that it is not in their control to change or improve their learning process or academic success (Raufelder et al., 2017; Sorrenti et al., 2019).

Beyond academic behaviors, LH in adolescents is shaped by a complex interplay of psychosocial and contextual factors within the school environment. Empirical evidence indicates that maladaptive parenting styles, such as overprotection and excessive criticism, alongside limited social support from peers and teachers, can foster the internalization of helpless attitudes (Margolis and McCabe, 2006; Sorrenti et al., 2019). Adolescents exposed to repeated academic failure, compounded by unsupportive social interactions, are more likely to develop maladaptive attributional styles characterized by persistent feelings of incompetence and tendencies toward social withdrawal. Moreover, individual personality traits, particularly elevated levels of neuroticism, have been associated with increased susceptibility to LH. Adolescents high in neuroticism are more prone to emotional instability, negative affect, and maladaptive rumination in response to academic and social setbacks (Liu and Alloy, 2010; Mezulis et al., 2004). Contextual factors within the school setting, including highly competitive academic climates and rigid teacher expectations, may further exacerbate helplessness by diminishing students’ perceived autonomy and self-efficacy (Raufelder et al., 2017). Collectively, these psychosocial and contextual determinants influence both the emergence and manifestation of LH in school-aged adolescents, with far-reaching implications not only for academic engagement but also for emotional well-being and interpersonal functioning.

Consequently, these helpless students develop a negative attitude toward school, leading to a withdrawal of school engagement, motivation and tasks (Filippello et al., 2020; Filippello et al., 2018). Moreover, students with LH tend to experience negative emotions, such as frustration, anger, stress, or anxiety, at the prospect of attending school (Filippello et al., 2020; Sorrenti et al., 2019), showing the relationship between LH and the School Attendance Problems (SAPs; Sorrenti et al., 2019; Sorrenti et al., 2016). SAPs has emerged to encompass a wide range of problems related to school attendance, like school refusal, school phobia, truancy, school withdrawal, etc. (Heyne et al., 2019; Kearney, 2016), due to the inconsistent terminology and definitions across studies (Heyne et al., 2019).

As multi-level approaches to address SAPs gain traction, the development of reliable assessment tools for SAPs has become increasingly critical (Gonzálvez et al., 2021). The *School Refusal Assessment Scale-Revised* (SRAS-R; Kearney, 2002) is the most widely used instrument, validated in multiple countries (e.g., US, Germany, France, Italy, UK, Turkey, Netherlands, and Spain), of which the combination of high and low scores across its four dimensions has identified distinct school refusal latent profiles (Delgado et al., 2019; Dube and Orpinas, 2009; Gallé-Tessonneau et al., 2019; Gonzálvez et al., 2020; Gonzálvez et al., 2023; Knollmann et al., 2013; Maynard et al., 2012; Pérez-Marco et al., 2024).

Taking into consideration the heterogeneity of causes that can explain school refusal behavior, in recent years new instruments have been published that aim to address new reasons not covered by the SRAS-R (Gonzálvez et al., 2021). One of them is the *Assessing Students’ Reported Reasons for School Non-attendance* (ARSNA; Havik et al., 2015) developed and validated in Norway for students of 6th to 10th grade, which evaluates four common school absenteeism reasons: Somatic Symptoms, Subjective Health Complaints, Unjustified Absences, and School Refusal. Despite its effectiveness, the ARSNA has been validated only in Spain, maintaining the factor structure of the original version and reporting adequate internal reliability indices (Martinez-Torres et al., 2024). Nevertheless, the identification of ARSNA profiles using latent profile analysis has not yet been applied.

There is a flawless gap in prior research on SAPs using the ARSNA dimensions in relation to LH. However, the numerous connections between these constructs should be acknowledged, given that multiple studies reveal associations between comparable aspects of SAPs and LH, even though the definitions and measurement of LH and SAPs differ across studies, as well as include populations with and without school attendance problems in school context.

Firstly, Somatic Symptoms and Subjective Health Complaints are highly associated with internalizing disorders (American Psychiatric Association, 2013), as well as LH (Sorrenti et al., 2019), because helpless students who refuse the school anxiously tend to feel their consequences on their own skin, like somatic symptoms or subjective health complaints, as a way of avoiding this school situations (Havik et al., 2015; Kearney, 2008; Kearney and Silverman, 1996; Thambirajah et al., 2008).

Therefore, it is shown statistically significant and positive correlations between LH and the appearance of Somatic Symptoms, such as feeling tired, trouble sleeping, indigestion, stomach pain or constipation, but among adult population (Ejdemyr et al., 2021). Moreover, Sorrenti et al. (2019) confirm the significant predictive capacity of LH on the appearance of the Somatic Symptoms of the internalizing disorders among children and adolescents. In terms of Subjective Health Complaints, Ree et al. (2014) found statistically significant and positive correlations between LH and Subjective Health Complaints, specifically high among male adults. However, regarding Somatic Symptoms and Subjective Health Complaints in children and adolescents, there is a clear lack of prior evidence.

In addition, students with low levels of school engagement tend to evaluate their academic life in a negative way, which can lead to school absenteeism, as well as they can present LH as a passive response to school problems (Mistry, 2024). Furthermore, this is confirmed by Raufelder and Kulakow (2021) and Raufelder et al. (2013), whom reported statistically significant correlations between LH and school belonging and school engagement, respectively, in a negative sense among adolescents. Moreover, Buzzai et al. (2021) affirm a significant and positive correlation between LH and School Alienation, which is the individual’s sense of detachment from school issues. For that reason, it is expected that helpless students tend to reject school tasks and have unjustified absences in school, due to the desire of avoiding school challenges (Buzzai et al., 2021).

Finally, regarding School Refusal, Sorrenti et al. (2016) showed in an Italian child sample the statistically significant and positive correlations between LH and each SRAS-R factor, where the dimensions with higher magnitude was the functional factors associated to anxious disorders, such as (FI) Avoidance of general school-related distress, (FII) Escape from adverse social and/or evaluative situations, and (FIII) Attention-seeking toward significant people, as well as the predictive capacity of LH on developing each SRAS factor, specially, FI and FII. Furthermore, the relevant relationship between LH and anxiety about school-related aspects (e.g., test anxiety, etc.) is well-established (Akca, 2011; Fincham et al., 1989; Raufelder et al., 2017), given that helpless students tend to experience anxious refusal to school to no deal with school tasks, due to their perception of lack of control over failures (Mistry, 2024; Raufelder et al., 2017). Likewise, in recent years, it has been observed that the levels of internalizing disorders have increased among the adolescent population, due to the health emergency situation experienced by COVID-19 (Fivush, 2025). Although there is no study that examines the level of learned helplessness among adolescents after this historical event, it can be expected that these helpless students now present higher levels of both learned helplessness and anxious disorders, and their possible somatization.

Previous research has not fully addressed the dimension of LH and the wide spectrum of reasons of school absenteeism, nor is there sufficient evidence regarding the links between latent school absenteeism profiles and LH, requiring more in-depth knowledge. Therefore, the primary objective of this study is to clarify the connection between LH and the ARSNA reasons of school non-attendance. Initially, the study aims to (1) identify latent profiles of school absenteeism based on ARSNA reasons. Although no prior theoretical evidence exists for ARSNA latent profiles, research on SRAS-R has found that the most common profiles include high or low scores across all factors, as well as mixed scores in different factors (Delgado et al., 2019; Dube and Orpinas, 2009; Gallé-Tessonneau et al., 2019; Gonzálvez et al., 2020; Gonzálvez et al., 2023; Knollmann et al., 2013; Maynard et al., 2012; Pérez-Marco et al., 2024). Consequently, three distinct profiles of school absenteeism are expected: (1) non-school absenteeism profile with low scores in all ARSNA reasons; (2) high school absenteeism with high scores in all ARSNA dimensions; and (3) mixed school absenteeism combining high and low scores in ARSNA dimensions.

Secondly, other purpose is to (2) carry out the analysis of the possible statistically significant differences between school absenteeism profiles as a function of the mean scores on LH. Despite the lack of previous research on the relationship of these latent profiles and LH, on the one hand, it is estimated that LH correlates positively and statistically significant with each dimension (Akca, 2011; Buzzai et al., 2021; Ejdemyr et al., 2021; Fincham et al., 1989; Raufelder and Kulakow, 2021; Raufelder et al., 2013; Ree et al., 2014; Sorrenti et al., 2016; Sorrenti et al., 2019); while, on the other hand, it is also expected that the high school absenteeism profile with high scores in ARSNA dimensions might score higher on LH, and the non-school absenteeism group would score lower on LH.

Finally, using variable-based perspective, this study aims to (3) establish the predictive capacity of LH of students belonging to the profile of high school absenteeism, and (4) its discriminative ability to identify individuals of the profile of high school absenteeism. According to Sorrenti et al. (2016) and Sorrenti et al. (2019), it is foreseen that LH predicts to belong to high school absenteeism group.

## 2 Method

### 2.1 Participants

A random cluster sampling was employed to select participants. The primary sampling units were geographical areas of the province of Alicante (North, South, Center, East, West). Educational centers served as secondary units, with a total of 8 public and private high schools selected. Finally, students’ groups constituted the tertiary units, with 4 groups randomly chosen from each high school. This sampling method produced a final sample of 759 Spanish students aged 12 to 18 (*M* = 14.95, *SD* = 1.82), comprising 328 boys, 419 girls and 12 students self-identifying as another gender. Table 1 presents the gender and age frequency distribution. No statistically significant differences across gender and age groups were observed, indicating a homogeneous distribution by gender and age (*x^2^* = 13.37; *p* = 0.34).

**Table 1:** Sample distribution by sex and age.

Gender	12 years	13 years	14 years	15 years	16 years	17 years	18 years	Total
Boys	35 4.6%	60 7.9%	55 7.2%	55 7.2%	58 7.6%	40 5.3%	25 3.3%	328 43.2%
Girls	37 4.9%	69 9.1%	70 9.2%	60 7.9%	76 10.0%	62 8.2%	45 5.9%	419 55.2%
Other	0 0%	0 0%	4 0.5%	2 0.3%	1 0.1%	2 0.3%	3 0.4%	12 1.6%
Total	72 9.5%	129 17%	129 17%	117 15.4%	135 17.8%	104 13.7%	73 9.6%	759 100%

### 2.2 Measures

*Assessing Reasons for School Non-attendance* (ARSNA; Havik et al., 2015). This self-report, consisted of 17 items, was used to assess students’ reasons for school absence on a 4-point Likert scale (0 = never, 1 = rarely, 2 = sometimes, 3 = very often). Students were asked to respond to each on a common question, ‘How often have you been absent from school in the last 3 months because…’. Responses span four factors: FI. *Somatic symptoms* (SS; e.g. ‘… you had a bad cold or flu?’); FII. *Subjective health complaints* (HC; e.g., “…you had a headache?”); FIII. *Reasons related to unexcused absences* (UA; e.g., “…you had a meeting?”); and FIV. *Reasons related to school refusal* (SR; e.g., “…you wanted to avoid unpleasant situations at school?”). Specifically, the ARSNA (Martinez-Torres et al., 2024) was used with internal consistency coefficients were 0.80, 0.85, 0.75 and 0.79 for each factor, respectively.

*Learned Helplessness Questionnaire* (LHQ). The LHQ (Sorrenti et al., 2015) is a self-reported instrument with 24 items assessing two sub-scales: *Learned Helplessness* (LH; e.g., “Does not respond with enthusiasm and pride when asked how one is doing on a school/academic task”) and *Mastery Orientation* (MO; e.g., “Tries to finish homework/assignments, even when they are difficult”). Students rate their argument with each statement on a 5-point Likert scale, from 1 (*not true*) to 5 (*absolutely true*). In this study, it is only assessed the dimension of LH, which reported acceptable reliability levels (*α* = 0.82).

### 2.3 Procedures

Following approval from the Ethics Committee of the University of Alicante (UA-2023-03-07) and the guide of Declaration of Helsinki (Rickham, 1964), the research objectives were presented to the school councils, and written informed consent was obtained from students’ parents and/or legal guardians. Students completed the self-reports questionnaires during the school day with anonymity and voluntary participation assured. During the 35-min assessment period, a researcher was present in the classroom to standardize the administration process.

### 2.4 Data analysis

Bivariate correlations between the four ARSNA factors and the LH were examined using Pearson’s correlation coefficients. According the Cohen’s *d*, the effect sizes were considered small with values between 0.10 and 0.29, moderate between 0.30 to 0.49, and large values over 0.50 (Cohen, 1988).

Secondly, a Latent Profile Analysis (LPA) was conducted, because it is considered optimal for classifying homogeneous groups of individuals, overcoming the limitations associated with *K*-means clustering (Schreiber, 2017). Model fit indices and criteria for selecting based on Song and Kim’s (2019) were applied to select the optimal class, prioritizing the lowest values of the Bayesian Information Criteria (BIC) and the Akaike Information Criteria (AIC), *p*-values below 0.05 for the Vuong-Lo–Mendell–Rubin Likelihood Ratio Test (LRT) and the Bootstrap Likelihood Ratio Test (BLRT), entropy values close to 1, and ensuring that each profile includes at least 25 participants (Tein et al., 2013). Theoretical interpretability of the profiles was also considered (Wang and Wang, 2020). For each factor, *z* scores were classified as low (scores < −0.5), moderate (scores between −0.5 and +0.5), and high (scores > + 0.5) (Gonzálvez et al., 2018).

An analysis of variance (ANOVA) was performed to identify differences between the school absenteeism profiles and the LH. *Post hoc* Bonferroni tests were applied to identify statistically significant differences among groups. Effect sizes were assessed using Cohen’s *d*, with values below 0.49 representing small effects, between 0.50 and 0.79 moderate effects, and above 0.80 large effects (Cohen, 1988).

A Binary Logistic Regression analysis was performed following the Wald forward stepwise method to examine the predictive capacity of LH for identifying students in *High School Absenteeism* profile. This predictive ability was estimated by the OR (Odd Ratio) statistic, where OR > 1 indicates a positive prediction, OR < 1 a negative prediction, and a value of 1 no prediction (De Maris, 2003).

Finally, ROC curves (sensitivity vs. specificity) were generated to assess the discriminative capacity of LH scores in detecting the likelihood of belonging to the *High School Absenteeism* profile. Interpretation of the area under the curve (AUC) value followed these criteria: between 0.75 and 0.90, the test is considered good; between 0.90 and 0.97, the discriminative ability is very good; and values between 0.97 and 1, discrimination is excellent (Martínez and Pérez, 2023). Sensitivity (true positive rate) is defined as the percentage of *High School Absenteeism* subjects who are correctly classified based on LH scores. Specificity (true negative rate) is the percentage of subjects not in this profile who are accurately identified by the LH scores. The Youden index was used to determine the cut-off point with optimal sensitivity and specificity.

Data analyses were conducted using IBM SPSS 28, Mplus 8.10 and MedCalc 19.

## 3 Results

### 3.1 Correlations between school absenteeism and learned helplessness

Table 2 presents the low to moderate correlations between School Absenteeism (SS, HC, UA, and SR) and LH. LH showed positive and statistically significant correlations with each ARSNA factor.

**Table 2:** Correlations between School absenteeism and learned helplessness.

Dimensions	SS	HC	UA	SR
Learned helplessness	0.25**	0.29**	0.12**	0.35**

SS, somatic symptoms; HC, subjective health complaints; UA, unjustified absences; SR, school refusal; *p < 0.05, **p < 0.001.

### 3.2 School absenteeism profiles

Table 3 provides fit indices for models with two to six profiles. Although the six-and five-profile models had the lowest AIC and BIC values, along with a *p* < 0.001 for the BLRT, they were rejected due to small cluster sizes (fewer than 25 participants) and suboptimal entropy. Similarly, the four-profile model was excluded, as it included a cluster with fewer than 25 subjects, despite its low AIC, BIC, and adjusted BIC values, as well as a significant BLRT (*p* < 0.001) and its entropy close to 1. The two-profile model was also dismissed, as it produced the highest BIC values. Consequently, the three-profile model was selected for its low AIC and BIC values, highest entropy (close to 1), and a significant BLRT (*p* < 0.001). All groups in this model were sufficiently representative of the sample and demonstrated superior classification utility, interpretability, and adequacy across all evaluated fit indices.

**Table 3:** Fit statistics for each latent profile model.

Models	AIC	BIC	BIC-adjusted	LRT *p*	LRT-adjusted	BLRT	Entropy	Size
2	7997.55	8057.77	8016.49	<0.001	<0.001	<0.001	0.71	0
**3**	**7613.78**	**7697.16**	**7640.01**	**<0.001**	**<0.001**	**<0.001**	**0.84**	**0**
4	7495.26	7601.80	7528.76	0.089	0.094	<0.001	0.83	1
5	7394.14	7523.83	7434.92	0.006	0.007	<0.001	0.82	2
6	7303.93	7456.78	7351.99	0.169	0.178	<0.001	0.81	2

AIC, Akaike Information Criterion; BIC, Bayesian Information Criteria; LRT, Vuong-Lo-Mendell-Rubin Likelihood-Ration Test; BLRT, Boostrap Likelihood Ratio Test.

**Figure 1:**
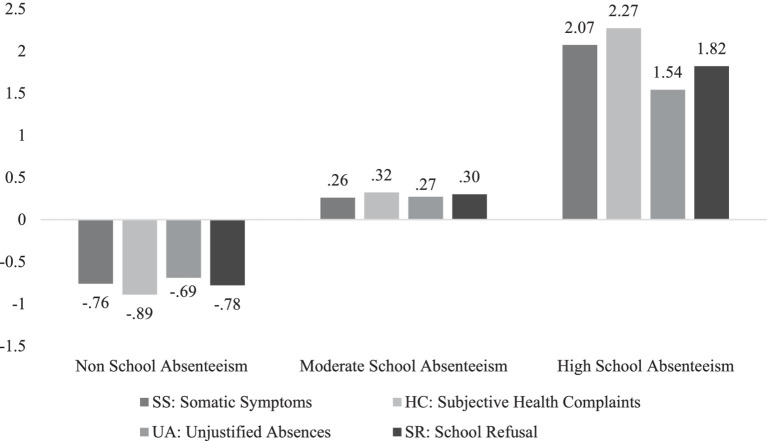
School absenteeism profiles.

Figure 1 illustrates the three-profile model of School Absenteeism. The first profile, named *Non-School Absenteeism*, included 266 subjects (35%), who scored low across all four factors (−0.76, −0.89, −0.69 and −0.78, respectively). The second group, *Moderate School Absenteeism*, comprises 453 subjects (59.7%) with low to moderate scores on each factor (0.26, 0.32, 0.27 and 0.30, respectively). Finally, the third profile, *High School Absenteeism*, consists of 40 participants (5.3%) who scored high on all ARSNA factors (2.07, 2.27, 1.54 and 1.82, respectively).

Table 4 presents the sociodemographic characteristics of the school absenteeism profiles. Regarding gender, it was observed that girls predominated across all three absenteeism profiles. Furthermore, the gender distribution among the profiles was reported to be homogeneous (*χ*^2^ = 1.23, *p* = 0.873). In terms of age, a disparity between the profiles was identified, which was supported by the Chi-square test (*χ*^2^ = 30.69, *p* = 0.002), indicating a non-homogeneous distribution across profiles. Specifically, students aged 16 predominated in Profile *non-School Absenteeism*, whereas Profiles of *Moderate School Absenteeism* and *High School Absenteeism* included a higher proportion of students aged 14 and 13, respectively. Concerning academic performance, students in Profile of *non-School Absenteeism* had passed all subjects, while in Profiles of *Moderate School Absenteeism* and *High School Absenteeism*, the majority of students had failed three or more subjects. This disparity was supported by the Chi-square test (*χ*^2^ = 37.76, *p* < 0.001), revealing statistically significant differences in the proportion of students passing or failing subjects according to their cluster membership. Lastly, regarding the relationship between cluster membership and parental educational level, statistically significant differences were found (*χ*^2^ = 19.42, *p* = 0.013). Specifically, in Profile of *High School Absenteeism*, a lower percentage of students (19.4%) reported that their father held a university degree, followed by Profile of *Moderate School Absenteeism* (27.2%), while Profile of *non-School Absenteeism* exhibited the highest percentage of students (39.8%) whose father had completed higher education.

**Table 4:** Sociodemographic characteristic of the school non-attendance profiles.

Profiles		Gender			Age							Academic performance			Parents’ studies				
		B	G	O	12	13	14	15	16	17	18	0S	1S	2S	NS	PE	SE	BV	UN
1	*N*	118	145	3	21	44	32	37	57	53	22	107	81	68	9	18	47	53	84
	%	44.4	54.5	1.1	7.9	16.5	12	13.9	21.4	19.9	8.3	41.8	31.6	26.6	4.3	8.5	22.3	25.1	39.8
2	*N*	195	250	8	47	73	92	72	75	47	47	112	134	194	14	48	96	107	99
	%	43	55.2	1.8	10.4	16.1	20.3	15.9	16.6	10.4	10.4	25.5	30.5	44.1	3.8	13.2	26.4	29.4	27.2
3	N	15	24	1	4	12	5	8	3	4	4	4	12	24	1	3	5	16	6
	%	37.5	60	2.5	10	30	12.5	20	7.5	10	10	10	30	60	3.2	9.7	16.1	51.6	19.4

Profile 1: non-school absenteeism; Profile 2: moderate school absenteeism; Profile 3: high school absenteeism; B: boys; G: girls; O: other; 0S: all approved signatures; 1S: 1/2 failed signatures; 2S: 3 or more failed signatures; NS: non-studies; PE: primary education; SE: secondary education; BV: bachiller/vocational education; UN: university.

### 3.3 School absenteeism profiles and learned helplessness

The ANOVA results indicate statistically significant differences between the School Absenteeism profiles and LH [Wilks’ Lambda = 0.87; *F*_(2, 756)_ = 29.45; *p* < 0.001; *n*_p_^2^ = 0.07], as shown in Table 5. The *High School Absenteeism* profile scored the highest on LH, followed by the *Moderate School Absenteeism* group, while *Non-School Absenteeism* profile had the lowest grades on LH.

**Table 5:** Means and standard deviations obtained by the four groups in the dimensions of learned helplessness.

Dimensions	Non-school absenteeism (*N* = 266)		Moderate school absenteeism (*N* = 453)		High school absenteeism (*N* = 40)		Statistical significance		
	*M*	DT	*M*	DT	*M*	DT	*F*_(2,756)_	*p*	η^2^
Learned helplessness	8.43	5.82	10.44	5.74	16.85	6.87	38.27	<0.001	0.09

Table 6 provides *post hoc* comparisons to identify statistically significant differences in LH scores across School Absenteeism profiles. All profile combinations showed statistically significant differences in LH scores. Effect sizes were moderate between *Non-School Absenteeism* and *Moderate School Absenteeism* profiles (*d* = 0.53), and large between *High School Absenteeism* profile and the groups of *Non-School Absenteeism* (*d* = 1.41) and *Moderate School Absenteeism* (*d* = 1.09).

**Table 6:** Cohen’s *d* value for *post hoc* values between cluster groups of school refusal profiles.

Dimensions		Non-SA – Moderate SA	Non-SA – High SA	Moderate SA – High SA
Learned helplessness	*p*	<0.001	<0.001	<0.001
	*d*	0.35	1.41	1.09

SA, school absenteeism.

### 3.4 Logistic regressions

Table 7 shows the results of the logistic regression analysis for the likelihood of belonging to the *High School Absenteeism* profile based on LH scores. The model accurately classified 94.7% of cases, with Nagelkerke’s *R*^2^ at 0.17. LH was a positive and significant predictor of belonging to the *High School Absenteeism* profile, with an OR of 1.18, indicating an 18% increase in the probability of belonging to this profile for each additional point on LH scale.

**Table 7:** Binary logistic regression for the probability of belonging to the High School Absenteeism profile as a function of the learned helplessness.

Variable		χ^2^	*R* ^2^	*B*	E. T.	Wald	*p*	*OR*	I. C. 95%
Learned helplessness	Correctly classified: 94.7%	46.16	0.17	0.17	0.03	42.36	<0.001	1.18	1.13–1.25
	Constant			−5.10	0.45	129.89	<0.001	0.01	

### 3.5 ROC curves

**Figure 2:**
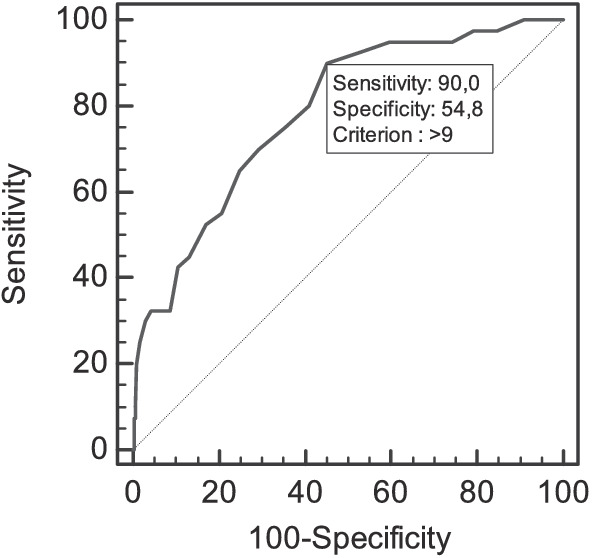
ROC curves for the discriminative capacity of the School Absenteeism scores over learned helplessness.

Finally, Figure 2 displays the area under the curve (AUC), indicating LH scores’ ability to distinguish individuals within *High School Absenteeism* profile. The AUC value at a cut-off score of 9 is 0.79 (95% CI = 0.75–0.81), which is significantly different above chance or a random ROC line (*p* < 0.001). Sensitivity reaches 90; specificity is 54.5 and the Youden index is 0.45, all suggesting that LH scores possess a solid discriminative capacity.

## 4 Discussion

This study seeks to explore the connection between SAPs and LH. Firstly, the latent profile analysis reported the existence of three student profiles of *low, moderate and high school absenteeism*. The resulting latent profiles cannot be compared with other studies, given the lack of previous related data identifying SAPs profiles using the ARSNA. However, the continuity of occurrence of high and low score profiles like the previous profiles based on the SRAS-R is observed (Delgado et al., 2019; Dube and Orpinas, 2009; Gallé-Tessonneau et al., 2019; Gonzálvez et al., 2020; Gonzálvez et al., 2023; Knollmann et al., 2013; Maynard et al., 2012; Pérez-Marco et al., 2024). Although it differs in the occurrence of mixed profiles with varying scores on the different factors assessed by the SRAS-R, which has not been observed with the ARSNA dimensions. This may mean that the distribution of latent profiles may be influenced not only by the characteristics of the sample, but also by the measuring instrument and the different factors measured (Çimşir and Ülker Tümlü, 2022) in each study.

Furthermore, the identification of three distinct absenteeism profiles partly reflects underlying psychosocial and contextual processes that shape students’ engagement with school. Specifically, the sociodemographic characteristics of the identified profiles indicate that variables such as age, academic performance, and parental educational level function as both individual and contextual factors that differentiate between opposing profiles, such as the *non-School Absenteeism* and *High School Absenteeism* profiles. More precisely, the *High School Absenteeism* profile predominantly consists of 13-year-old students who exhibit lower academic achievement and whose parents possess lower levels of educational attainment compared to those in the more adaptive profiles. Moreover, this maladaptive profile not only exhibited elevated levels of LH, but also experienced cumulative academic, interpersonal, and contextual risk factors that reinforce their disengagement from school. Prior research suggests that repeated academic failure and negative feedback can undermine students’ sense of competence and foster maladaptive attributional styles characteristic of LH (Margolis and McCabe, 2006; Liu and Alloy, 2010). These students tend to attribute their difficulties to stable and internal causes, which perpetuates feelings of incompetence and resignation.

In addition to academic difficulties, peer relationships and family context play a pivotal role in determining profile membership. Adolescents facing low peer acceptance, bullying, or poor teacher-student relationships often report diminished school belonging and heightened emotional distress (Raufelder et al., 2017), which consequently increases school absenteeism (Leduc et al., 2024). Such experiences not only erode social support but may also exacerbate LH by reinforcing the belief that personal efforts cannot alter negative interpersonal outcomes. Furthermore, family dynamics characterized by low parental support, overprotection, or excessive academic pressure have been linked to increased vulnerability to helplessness, further compounding school disengagement and refusal (Gubbels et al., 2019; Sorrenti et al., 2019).

Regarding the ANOVA results, adolescents with *high school absenteeism* profile evidenced the highest means on LH, confirmed by the large magnitude of the effect size between the *high school absenteeism* profile with the profiles *non-school absenteeism* and *moderate school absenteeism* (*d* = 1.41; *d* = 1.09, respectively). This may be due to students’ own perceived inability to respond to problems and challenges in order to improve and succeed academically (Raufelder et al., 2017; Sorrenti et al., 2019). This, in turn, results in young people who reject school developing a passive response to problems and a belief that they cannot control this helpless attitude, thus developing LH. Moreover, given the continuous succession of school failures, students have developed a school refusal because of the anxiety to attend there or even the belief of no control over failure. Therefore, these school refusers end up avoiding school challenges or difficult tasks (Buzzai et al., 2021; Mistry, 2024; Raufelder et al., 2017), and even procrastinating, as an avoidance response to unpleasant school situations (Wang, 2020).

The above results are supported by those derived from the variable-centered analysis. Logistic Regression showed that LH is a positively and significantly predictor of belonging to the *high school absenteeism* profile. In the same vein, the ROC curves revealed that the discriminative capacity of the LH is good, allowing the correct classification of 79% of the students belonging to the *high school absenteeism* profile, despite not being able to compare these results with previous studies given the lack of research on the subject. The cut-off point that best discriminates subjects who do or do not belong to the *high school absenteeism* profile is 0.90 for LH. In this case, sensitivity level above 0.90 is obtained, indicating that this cut-off point allows us to correctly classify the 90% of the *high school absenteeism* profile subjects (sensitivity), and with a lower percentage (54.8%) for those who do not actually belong to the *high school absenteeism* profile (specificity) (Martínez and Pérez, 2023).

Consequently, the results of the ANOVA and Logistic Regression analyses underscore that LH significantly predicts membership in the *high school absenteeism* profile. However, this association should be viewed not solely as an outcome of academic experiences, but as part of a broader psychological pattern. LH is a multidimensional construct with cognitive (pessimistic attributional style), emotional (low self-efficacy, anxiety), and behavioral (avoidance, passivity) components (Abramson et al., 1989; Seligman, 1975). School absenteeism could be seen as a behavioral manifestation of these internal processes, but LH likely impacts other areas such as academic motivation, emotional regulation, and social interactions. Thus, interventions targeting only absenteeism without addressing underlying cognitions and emotions related to LH may have limited efficacy. Moreover, the ROC curves showing the good discriminatory capacity of LH supports its relevance as a potential screening marker in educational settings. Identifying students at risk of SAPs through their helplessness levels could facilitate early interventions.

Collectively, the proposed hypotheses have been confirmed by the above results, in line with preliminary studies in the field of SAPs and LH. Thus, the results found in this study provide sufficient evidence to suggest that the SAPs assessed by the ARSNA may be positively and significantly associated with LH. Certain aspects of SAPs, such as Somatic Symptoms, Subjective Health complaints or School Refusal, may be more strongly influenced by LH due to the anxiety-based factors. However, all of these ARSNA dimensions have been observed to correlate with LH.

This study presents certain limitations that should be acknowledged. Firstly, data collection relied on a self-report method, which could introduce biases related to social desirability. Secondly, caution is warranted when attempting to generalize these findings to different age groups or cultural contexts, due to the characteristics of community Spanish sample. Consequently, it could be beneficial to replicate this research in various countries to enable comparisons and explore whether cultural differences influence the relationship of SAPs and LH, as well as in specific groups (e.g., students with diagnosed school attendance problems). Moreover, a further limitation lies in the measurement of school absenteeism. Although absenteeism levels were classified into low and high groups through Z-scores and latent profile analysis, the exact number of unexcused absences could not be provided due to the absence of an established consensus on cut-off points. Future studies should aim to define standardized thresholds or include more precise measures of absence frequency.

With regard to the construct of LH, several limitations also should be recognized. On the one hand, it is important to recognize that LH is a multidimensional construct, with multiple theoretical frameworks and instruments available to assess and explain this students’ self-perception. In the present study, only one measure, the Learned Helplessness Questionnaire (LHQ), was employed. Therefore, future research would benefit from examining the same sample using alternative theoretical models and assessment tools of LH, in order to enable comparative analyses and gain a more comprehensive understanding of how different conceptualizations of LH manifest within the same population. On the other hand, an additional important limitation is the omission of the Mastery Orientation (MO) subdimension from the analysis of the Learned Helplessness Questionnaire (LHQ). This omission restricts the ability to compare how students from the most maladaptive and the most adaptive school absenteeism profiles respond to academic challenges. Furthermore, future studies could categorize high and low levels of LH among students based on empirically established cut-off points, in order to more accurately and rigorously determine the extent to which this psychological condition is present within the student population. Lastly, due to the study’s cross-sectional design, casual inferences between variables cannot be established. Therefore, future research should incorporate longitudinal data to examine students’ school refusal across different stages of their education.

Despite its limitations, this research brings valuable new insights to the field of Educational Psychology. Specifically, it clarifies the relationship between ARSNA school absenteeism factors and the LH through a dual approach, combining variable analysis (correlations, logistic regression and ROC curves) and person-oriented analysis (latent profile analysis and ANOVA). Additionally, it is the first study to employ, on the one hand, the latent profile analysis with ARSNA dimensions to identify the groups of school absenteeism; and, on the other hand, ROC Curves analysis to assess the discriminative power of LH to distinguish students of *high school absenteeism* profile. It is also worth underlining the need to recognize among educational professionals that the intervention against LH can be a common and positive initiative to reduce SAPs in adolescents, as well as the sense of school exclusion and poor teacher-student relationships are risk factors that may increase the likelihood of developing LH among school refusers (Raufelder and Kulakow, 2021). Understanding how LH and school non-attendance are related might help schools create focused treatments, such teacher training, mental health assistance, and flexible learning strategies, to address underlying issues and foster resilience. Furthermore, it is worth mentioning that measuring this aspect of students’ mental health, such as LH, may improve the usefulness of the assessment in students with SAP who would have represented significant heterogeneity in their profiles, and their subsequent design of personalized intervention. These results can also guide community collaborations, parental involvement tactics, and legislative modifications to foster a positive atmosphere that minimizes absenteeism and its psychological effects, apart from to design intervention programs to reduce school absenteeism rates and, simultaneously, increase learned helplessness levels. For that reason, the use of Cognitive-Behavioral therapy and/or strategies (i.e., imaginary strategies, mindfulness, cognitive reconstruction, progressive exposure, etc.) was confirmed, due to its effectiveness in the interventions against school absenteeism (Pérez-Marco et al., 2025) and learned helplessness (Aslam and Bano, 2019), separately, in order to improve the students’ overall wellbeing. In this sense, possible future studies may develop a single program that allows the treatment of both variables through the same type of therapy.

## Data Availability

The raw data supporting the conclusions of this article will be made available by the authors, without undue reservation.
